# Detecting Impending Stroke From Cognitive Traits Evident in Internet Searches: Analysis of Archival Data

**DOI:** 10.2196/27084

**Published:** 2021-05-28

**Authors:** Sigal Shaklai, Ran Gilad-Bachrach, Elad Yom-Tov, Naftali Stern

**Affiliations:** 1 Institute of Endocrinology, Metabolism and Hypertension Tel Aviv Sourasky Medical Center Tel Aviv Israel; 2 Sackler Faculty of Medicine Tel Aviv University Tel Aviv Israel; 3 Sagol Center for Epigenetics of Aging and Metabolism Tel Aviv Sourasky Medical Center Tel Aviv Israel; 4 Faculty of Bio-Medical Engineering Tel Aviv University Tel Aviv Israel; 5 Edmond J Safra Center for Bioinformatics Tel Aviv University Tel Aviv Israel; 6 Microsoft Research Herzeliya Israel; 7 Faculty of Industrial Engineering and Management Technion Haifa Israel

**Keywords:** search engines, diagnosis, screening, stroke, risk, internet, trend, infodemiology, archive, prospective, algorithm

## Abstract

**Background:**

Cerebrovascular disease is a leading cause of mortality and disability. Common risk assessment tools for stroke are based on the Framingham equation, which relies on traditional cardiovascular risk factors to predict an acute event in the near decade. However, no tools are currently available to predict a near/impending stroke, which might alert patients at risk to seek immediate preventive action (eg, anticoagulants for atrial fibrillation, control of hypertension).

**Objective:**

Here, we propose that an algorithm based on internet search queries can identify people at increased risk for a near stroke event.

**Methods:**

We analyzed queries submitted to the Bing search engine by 285 people who self-identified as having undergone a stroke event and 1195 controls with regard to attributes previously shown to reflect cognitive function. Controls included random people 60 years and above, or those of similar age who queried for one of nine control conditions.

**Results:**

The model performed well against all comparator groups with an area under the receiver operating characteristic curve of 0.985 or higher and a true positive rate (at a 1% false-positive rate) above 80% for separating patients from each of the controls. The predictive power rose as the stroke date approached and if data were acquired beginning 120 days prior to the event. Good prediction accuracy was obtained for a prospective cohort of users collected 1 year later. The most predictive attributes of the model were associated with cognitive function, including the use of common queries, repetition of queries, appearance of spelling mistakes, and number of queries per session.

**Conclusions:**

The proposed algorithm offers a screening test for a near stroke event. After clinical validation, this algorithm may enable the administration of rapid preventive intervention. Moreover, it could be applied inexpensively, continuously, and on a large scale with the aim of reducing stroke events.

## Introduction

Cerebrovascular disease is a leading cause of mortality and disability. Further, with the worldwide aging of the population, the rise in stroke mortality is only second to that attributed to heart disease [1]. The overall care of stroke survivors comprises an immense social and economic burden due to the high incidence (~30%) of residual cognitive and physical disabilities [2]. Hypertension, atrial fibrillation, hyperlipidemia, smoking, obesity, and diabetes comprise the major treatable risk factors that underly the occurrence of most stroke events [3]. With the exception of obesity and smoking, these conditions are often asymptomatic and therefore can be missed with treatment deferred for years, unless incidentally detected, clinically suspected, or realized during an acute vascular event. Even when subjects or physicians are aware of these conditions, treatment is not consistently initiated and is often insufficient or discontinued [4,5] due to patient or health care system neglect. Effective means now exist that can substantially lower the rate of stroke shortly after their implementation, as best exemplified by the early benefits of immediate treatment with anticoagulants for atrial fibrillation and blood pressure–lowering drugs for hypertension [6,7]. Therefore, the timely detection of an impending stroke might open a time window of opportunity for immediate medical intervention that can save lives and prevent poststroke disability. To our knowledge, the only risk calculator for impending stroke has been constructed in subjects who have already experienced a previous single stroke, aiming to predict the 90-day risk of recurrent stroke [4], but not for first-ever stroke events.

Computer-based analysis of electronic health records has the potential to provide major novel insights of benefit both to specific individuals in the context of personalized medicine as well as on the level of population-wide health care and policy [8]. However, such analysis mostly relies on traditional risk factors that have already been at the core of daily practice for several decades. The most widely accepted models for stroke prediction, such as those of the American College of Cardiology/American Heart Association cardiovascular disease risk algorithm and the Framingham risk score actually offer a more general predictive target, usually referred to as an “event,” which includes an overall risk of occurrence of stroke and other vascular events such as myocardial infarction, death from coronary heart disease, congestive heart failure, incident angina, or intermittent claudication [9]. Further, although these risk profiling models provide clinically critical information in that they calculate the likelihood of an event in the near decade, they have not been constructed to evaluate the risk of an impending short-term cardiovascular threat.

An alternative approach for predicting stroke relies on identification of covert cerebrovascular disease, which often precedes stroke. Covert cerebrovascular disease is associated with subtle cognitive and motor deficits, and increased risk for stroke and further cognitive decline [10,11]. Currently, this can be diagnosed by either neuroimaging or in-person assessment using conventional cognitive screening tests; however, both tools are costly, not effective on the population as a whole, and not continuous [12].

The way that an individual interacts with their computer has been suggested to represent an as-yet unexploited means of assessing everyday cognition [13]. Computer interaction requires motor, language, and executive functions; ability to operate the keyboard and mouse; comprehend and create text; plan; and focus attention. Routine use such as querying of search engines has been shown to correlate with parts of standard cognitive tests, and may thus be used as a continuous, unobtrusive, inexpensive monitoring application [13]. More broadly, internet data have been shown to be a useful source for studying health and improving public health. This has become an active field of research, known as infodemiology, a term coined by Eysenbach [14,15]. Due to their ubiquitous nature and anonymity [16], search engine queries have been shown to be useful for screening for a variety of diseases, including Alzheimer disease [17], Parkinson disease [18], several types of cancer [19-21], diabetes [22], and eating disorders [23]. Similarly, social media postings have been shown to change prior to emergency department visits [24].

In this study, we evaluated the possibility that subjects disclosing the fact that they have experienced a stroke can be singled out from subjects without such a self-declared history via their internet-based communications prior to the occurrence of the stroke. If prestroke patients show unique alterations in communication features, this could potentially be utilized as a “last minute” alarm to seek prompt medical help, with the hope that an impending stroke can be prevented.

## Methods

### Data

Dataset 1 comprised all queries submitted to the Bing search engine by people in the United States during a 2-year period beginning July 2017. The data comprised the query text, the time and date, an anonymous user identifier, and data on the interaction between the user and the search results page.

To identify a “patient” cohort, we found users who queried the search engine for phrases that indicated that the user underwent a stroke, such as “I had a stroke” or “I was diagnosed with a stroke” (referred to as the “reference query”). Specifically, we defined the reference queries to be queries containing one of the phrases “I had,” “I experienced,” or “I suffered” *and* one of the phrases “stroke,” “cerebrovascular accident,” “CVA,” “cerebrovascular event,” “transient ischemic attack,” “brain infarction,” “brain ischemia,” and “cerebral ischemia.”

Additionally, we required that users were active for at least 10 days before and after this query, made at least 50 queries, and had no more than 3 periods of inactivity of 24 hours or more in the 60 days prior to the reference query. We also required that in the 60 days prior to the reference query, the user did not issue queries for between 1 and 10 days, which, we hypothesized, represented the time that the user was hospitalized.

We chose several populations as controls, including a random sample of people aged 60-64, 65-74, or 75 years or older, and random samples of people aged 60 years or older who queried one of the following conditions (separately): depression, migraine, B12 deficiency, heart attack, hypothyroidism, surgery, atrial fibrillation, hypertension, and Alzheimer disease. Table 1 lists the terms used to identify these conditions. Ages were determined according to information provided by users at the time of registration to Bing.

The “reference query” for the control populations was chosen as a random query from their queries (for people selected by their age) or the first query mentioning the condition of interest (for those who queried having experienced one of the control conditions above). All control populations were filtered for the same activity levels (as described above) as the patient cohort.

Dataset 2 was similar to Dataset 1, but was taken from July 2019 to June 2020 and was limited to users who were aged 60 years or more.

**Table 1:** Number of users in the control cohorts according to various conditions and the search terms used to identify the group.

Cohort	Terms or criteria used for identification	Users, N
60-64 years old	According to information provided at registration to Bing	108
65-74 years old	According to information provided at registration to Bing	82
>75 years old	According to information provided at registration to Bing	38
Depression	depression, depressed, ssri, citalopram, celexa, escitalopram, lexapro, fluoxetine, prozac, fluvoxamine, luvox, paroxetine, paxil, sertraline, zoloft, atomoxetine, Strattera, pristiq, desvenlafaxine, cymbalta, duloxetine, levomilnacipran, fetzima, milnacipran, ixel, savella, dalcipran, toledomin, sibutramine, meridia, tramadol, ultram, venlafaxine, effexor	85
Migraine	migraine, metoprolol, Lopressor, valproate, Depakote, epilim, topiramate, Topamax	66
B12 deficiency	B12	48
Heart attack	heart attack, acute myocardial infarction	101
Hypothyroidism	hypothyroidism, levothyroxine, levoxyl, synthroid, tirosint, unithroid	59
Surgery	Prostatectomy, hip replacement, surgery	369
Atrial fibrillation	atrial fibrillation, anticoagulants, eliquis, apixaban, xarelto, rivaroxaban, pradaxa, dabigatran, lixiana, edoxaban, coumadin, warfarin, antiarrythmic, amiodarone, procor, cordarone, sotalol, flecainide, tambocor, propafenone, rythmol, dronedarone, multaq, dofetilide, tikosyn	86
Hypertension	hypertension, angiotensin receptor blocker, irbesartan, telmisartan, candesartan, atacand, valsartan, diovan, losartan, cozaar, losardex, olmesartan, benicar, ace inhibitor, benazepril, lotensin, captopril, cilazapril, enalapril, enaladex, fosinopril, lisinopril, ramipril, moexipril, cardiotensin, perindopril, quinapril, accupril, trandolapril, mavik, mineralocorticoid receptor antagonists, spironolactone, aldactone, carospir, eplerenone, inspra, diuretics, chlorthalidone, calcium channel blocker, amlodipine, norvasc, katerzia, diltiazem, cardizem, matzim, taztia, tiazac, verapamil, verap, alpha blocker, doxazosin, cardura, cadex	78
Alzheimer disease	((I or my) AND (forgetting, misplacing, can't find, forget name)) OR Alzheimer OR dementia	75

### Data Analysis

Each user was represented through the average values of several attributes and the SDs thereof, computed for all queries within a 2-day moving-window period. These attributes were chosen to represent cognitive ability [13,25,26], activity routine, and indications related to risk factors for stroke. The list of attributes is as follows: (1) number of words per query; (2) time of day; (3) the likelihood of the query string in the entire population; (3) number of new words in the query, compared to all previous queries by the user; (4) time since previous session; (5) number of queries per day and per hour; (6) time from the display of the results to the first click on a result; (7) farthest link clicked by the user; (8) use of automatic spelling correction; (9) whether the query was submitted by this user in the past; and (10) specific keywords in the query, including personal references, mention of relevant symptoms, or mention of certain medicinal drugs.

A session was defined as a continuous period of querying, followed by a break of 30 minutes or longer. Additionally, we quantified the ratio or each attribute to its values for the same user 10 days before. As noted above, we assumed that the gap in user queries just before the reference query was likely due to hospitalization following a stroke. Therefore, we defined the time of the last query prior to the gap as time zero for each user and calculated the time of each user’s query relative to time zero. We refer to this as the “relative time” of a query. Patterns were augmented with the average value of each attribute within the relative time of –150 to –120 days.

Unless otherwise stated, we used all data from 30 days prior to a gap in the queries, which was followed by the reference query and up to that gap, to build a random forest model with 1000 trees to distinguish the patient cohort from each of the control cohorts. Ten-fold cross-validation at the user level was applied to reduce the likelihood of overfitting. All processes were performed using Matlab 2019. This study was approved by the Institutional Review Board of Technion, Israel Institute of Technology.

## Results

### Separation of the Stroke Cohort From Control Cohorts

**Figure 1:**
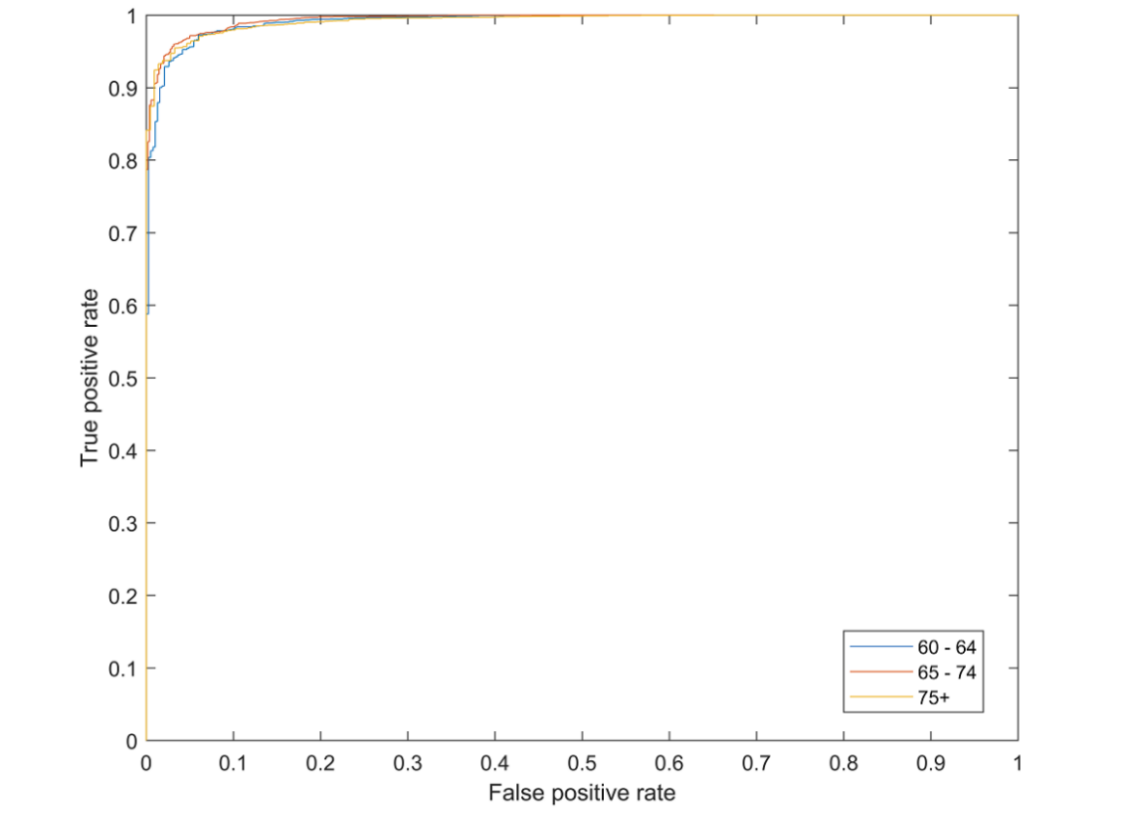
Receiver operating characteristic curve for distinguishing users who underwent stroke from controls for people of different ages.

We identified 285 users in the patient cohort; Table 1 shows the number of users in each of the control cohorts. Figure 1 shows the receiver operating characteristic (ROC) curve [27] for distinguishing the patient cohort and the control by age cohorts. These data comprise only the queries prior to the gap preceding the reference query. The ROC curve showed that excellent differentiation between the patient and control cohorts is possible.

**Figure 2:**
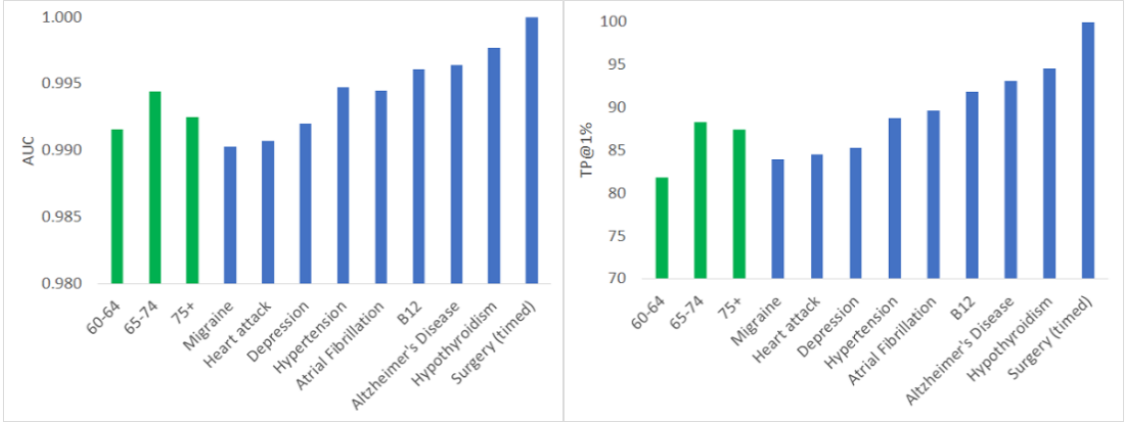
Area under the receiver operating characteristic curve (AUC, left) and the true positive rate at a 1% false-positive rate for separating the patient cohort from each of the control cohorts.

Figure 2 shows the area under the ROC curve (AUC) [27] and the true positive rate at a 1% false-positive rate for separating the patient cohort from each of the control cohorts. Very good performance was reached for all conditions. In addition, some cardiovascular diseases (heart attack, hypertension, and migraine) and certain mental diseases (depression) appeared to be harder to separate from stroke.

The best attributes for separating the stroke cohort from the other cohorts were estimated by finding the increase in prediction error if the values of that variable were permuted for the test observations, using the appropriate Matlab function (OOBPermutedPredictorDeltaError). The five most important attributes for separating the stroke cohort from each of the control conditions were (in descending order of the number of conditions in which they appeared in the top 5): average query likelihood, SD of the query likelihood, average number of queries per session, average number of spelling mistakes, and average number of repeated queries.

Although the model does not use specific vocabulary in identifying people at risk, it is nevertheless interesting to study the differences in the vocabulary used by the patient cohort and the control groups, and how the vocabulary changes after the stroke event.

Risk factors for stroke include high blood pressure, hypertension, diabetes, insulin, heart disease, cardiomyopathy, heart failure, atrial fibrillation, blood clot, smoking, cocaine, amphetamines, sickle cell disease, vasculitis, bleeding, overweight, stress, and cholesterol. Among people in the patient cohort, 32.2% queried one or more of these risk factors prior to day zero and 56.4% queried for these terms after day zero. Among people 60 years or older in the control cohort, the corresponding percentages were 4.3% and 4.6%.

Medical drugs used to treat survivors of stroke include plavix, clopidogrel, aspirin, dipyridamole, pradaxa, dabigatran, eliquis, apixaban, xarelto, rivaroxaban, atorvastatin, lipitor, simvastatin, simovil, rosuvastatin, crestor, pravastatin, and pravachol. Among people in the patient cohort, 12.5% queried one or more of these drugs prior to day zero and 28.2% queried for these terms after day zero. Among people 60 years or older in the control group, the corresponding percentages were 0.7% and 0.8%. We assume that some of the people mentioning the drugs in the control group and prior to the day of stroke in the treatment group did so because they received these drugs due to their being in high-risk groups for the disease.

These two results suggest that users in the patient cohort did indeed undergo a stroke at the estimated date.

### Prospective Cohort

We repeated the same analysis with a prospective cohort comprising users in Dataset 2. Since relatively few people had mentioned that they experienced stroke and were over 60 years of age, our labels were whether or not a user asked about stroke more than a given number of times. This is a weaker proxy than experiential queries, but is nevertheless known to be a good proxy [28]. The control group consisted of all other users.

Our classification model was constructed using data from Dataset 1. Data for the prospective cohort comprised queries until 14 days before a gap followed by the first mention of a stroke.

**Figure 3:**
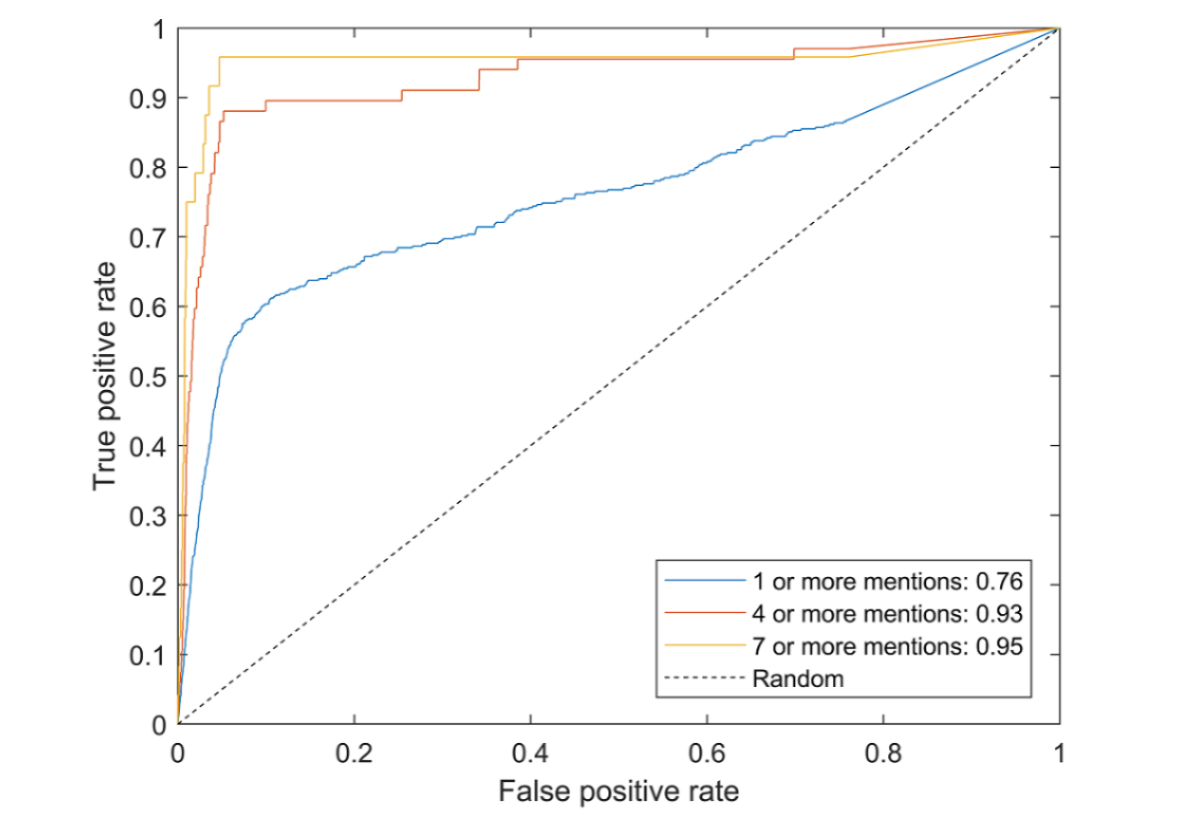
Receiver operating characteristic curve for the prospective cohort; the area under the curve (AUC) values for the different settings are presented in the legend.

Figure 3 shows the ROC curve for the resulting experiment, demonstrating the ability to detect people who will, in future, ask multiple times about stroke.

### Temporal Change Prior to the Indication of Stroke

We used the attributes derived for people in the patient cohort from Dataset 1 to examine the ability to localize stroke in time. Toward this end, we considered two time constants, N_1_ and N_2_, to define the negative examples as data from before N_1_ days prior to time zero and positive examples as data from N_2_ days prior to time zero until time zero, essentially training a classification model to predict if a user will undergo a stroke in the next N_2_ days.

**Figure 4:**
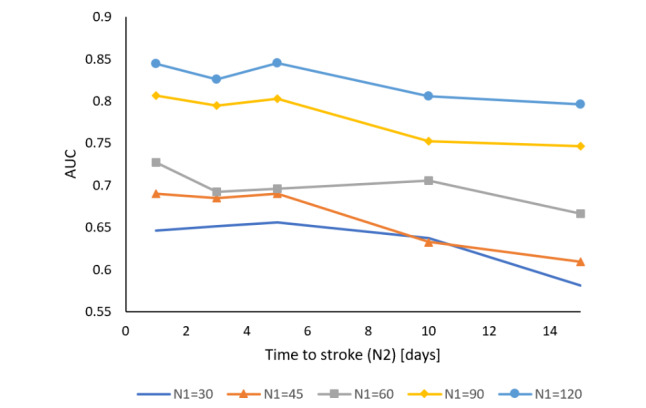
Area under the receiver operating characteristic curve (AUC) as a function of N_2, the number of days ahead for which the prediction is made. Different curves show different values of N_1, the time prior to stroke that is taken as nonstroke dates.

The model was a random forest model with 1000 trees. Ten-fold cross-validation stratified by users was utilized in the estimation of model performance. Figure 4 shows the AUC as a function of N_1_ and N_2_. Relatively accurate classification could be obtained for the stroke localization task, especially for times close to the event. For example, Figure 4 shows that separation between data coming from 120 or more days before the stroke and data coming from 14 days before the event can be classified with AUC≥0.8. Moreover, two effects are visible. First, as the separation between N_1_ and N_2_ increases (N_2_ is larger), performance is improved. Second, the closer to the date of estimated stroke, the better the prediction. We hypothesize that the signal of stroke becomes stronger closer to the time of the stroke and thus negative training data taken close to the date of stroke confuse the model. Second, by limiting the positive examples to those near the actual stroke date, the data contain stronger indications of impending stroke, making the classification more accurate.

**Figure 5:**
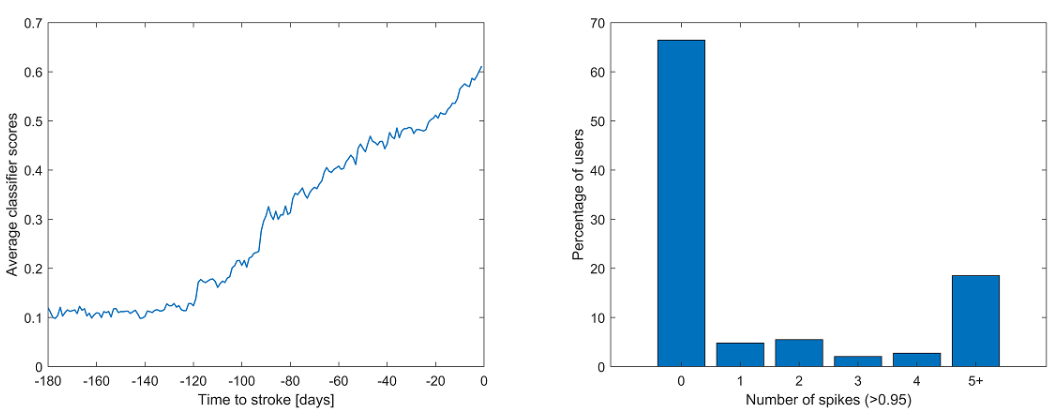
Average classifier scores as a function of the time to estimated stroke date (left) and the number of times that each user had classifier scores greater than 0.95 (right) in the period between 120 days prior to the estimated stroke date and the day before.

To further investigate this hypothesis, we examined times close to the estimated stroke time with respect to the predicted classifier scores (with N_1_=90, N_2_=5). Figure 5 shows the average classifier scores (in the range of 0 to 1) as a function of the time relative to the estimated stroke time. The classifier scores began to rise around 120 days before the estimated stroke day. In addition, Figure 5 shows the number of days during the prior 120 days and until the day before the estimated stroke day on which users had a score of over 0.95. A significant percentage of users had 5 or more days on which their scores were extremely high. Taken together, these findings suggest that significant alterations can be recognized prior to the date of estimated stroke.

## Discussion

### Principal Findings

Our findings suggest that among internet users, stroke events are preceded by alterations in communication patterns that have been previously shown to reflect aspects of cognitive function [29]. We used an unusual break in the query stream prior to the first mention of a stroke as the likely time of the clinical cerebrovascular event. Assuming that our presumed time of stroke correctly approximates the time of the real event, the internet-derived signal of an impending stroke tends to gain strength as the time of the stroke approaches. If monitored continuously, not only the signal per se but also its persistence and intensification with time might comprise a useful aid in attempted identification of an impending stroke. We expect that these or similar changes in pattern will be present in subjects at risk for stroke prior to a clinically verified stroke. Clearly, following the present hypothesis-forming analysis, the usefulness of the prediction model described here must be validated, and perhaps improved in a clinical setting among internet users who underwent a documented stroke.

Since stroke is mostly a condition affecting the older population segments, the complexity of multiple confounding morbidities comprised a real challenge to our study. We therefore included four types of control groups: (1) subjects not mentioning any health problems; (2) subjects mentioning acute nonvascular medical conditions (ie, surgery); (3) subjects who mentioned an acute vascular event, acute myocardial infarction with risk factors that are nearly identical to those of stroke; and (4) a variety of chronic conditions, which are relatively common in older subjects and may affect cognition, including hypertension, depression, vitamin B12 deficiency, and hypothyroidism. The model developed in this study performed well against all comparator groups, but showed better distinction from surgery and acute myocardial infarction, with more limited power to separate the stroke signal from that generated by hypertension. However, even in the weakest comparisons, the performance via the ROC analysis was very high, with an AUC in excess of 0.985 for all comparators. To place this performance in the proper context, the revised Framingham Stroke Risk Score, which is based on traditional risk factors and predicts a 10-year risk of stroke, offers an AUC of ~0.71 at best [3].

This study cannot uncover the mechanism underlying the evolution, prior to stroke, of escalating cognitive signals that can be detected via examination of an internet communication pattern. A recognizable stroke could be preceded by showering of microemboli from the arterial tree or the heart; worsening of brain small-vessel disease by uncontrolled hypertension and/or hyperlipidemia; or former smaller, clinically unrecognized microstrokes such as in the case of paroxysmal atrial fibrillation or in situ thrombi with cycles of partial resolution and expansion, all subtly impairing cognitive function prior to a major event. Such putative mechanisms would be hard to verify/exclude in the absence of solid clinical, biochemical, and imaging information.

There appears to be a three-fold advantage to an internet-based app for predicting impending stroke such as that presented here. First, it has the potential of serving as an alarm signal, which becomes gradually more intense as the time of the actual stroke approaches, beginning at 120 days to a few days before the stroke. This offers a time window of opportunity to act swiftly, identify specific underlying conditions that predispose to stroke, and apply preventive measures. This concept potentially generates a concrete and immediate alarm, vastly remote from calculating a general “sometime in the next decade” warning, which is often ignored. Second, if indeed validated in the clinical setting, its predicted overall performance and accuracy are considerably higher than those of solidly established traditional tools. Lastly, this algorithm can be applied in an inexpensive manner on a large scale and ongoing basis, similar to a computer virus detection program that runs in the background, reducing costly screening, morbidity, and mortality [17].

### Limitations

This study has important inherent limitations. First, the assumption that subjects who declared that they had a stroke indeed experienced some form of true cerebrovascular event lacks actual clinical documentation. However, we provide indirect evidence that the identification was likely correct, including a period of “internet silence” suggesting hospitalization; queries for risk factors and relevant drugs, which increased substantially in the stroke cohort after the event; increase in classifier scores leading up to the estimated stroke time; and the attributes used to separate the stroke cohort from the controls. Moreover, many other people who underwent a stroke event did not identify themselves as having experienced it. Those people would not have been included in our cohort and might therefore introduce a bias toward specific populations [30].

Second, since the terms used to trap the occurrence of stroke were selected by lay people, the precise nature of the event is impossible to determine, neither is the ratio among various forms of cerebrovascular events such as true stroke vs transient ischemic attack. The requirement for a minimal period of internet “absenteeism” as an inclusion criterion would likely, but not completely, exclude very minor events. As is the case for the occurrence of stroke itself, the existence of the comparator conditions such as acute myocardial infarction completely depends on wording in queries and lack actual medical evidence.

Third, our analysis only applies to presumed stroke survivors who could return to use the internet. Therefore, it does not apply to individuals who did not survive the stroke or had an unfortunate clinical course that precluded further internet communications after the stroke; thus, our analysis was likely not applied to the most severe forms of stroke. Although it is possible that subjects that suffered fatal or severely disabling stroke would have no prestroke warning fingerprints in their communications, it is equally reasonable to assume that their prestroke signals could be even stronger. Moreover, the actual use of drugs is likely higher than their mention in communications and could also refer to people other than the subjects under study themselves.

We also note that the identification of people in the control condition, although supported by precedent [28], does not preclude people who queried for events that happened in the past, because they were asking about other people or those who were asking out of general interest. However, as the cited literature suggests, they are a reasonable proxy for such a condition cohort.

### Conclusions

We have developed an internet-based detection model that retrospectively identified subjects who later developed stroke according to self-reports. The usefulness of this system in real-life patients awaits validation in a clinical setting where this model must be tested against accurate diagnoses, information on stroke risk factors, comorbidities, drugs, and outcome. We put forth the vision, which requires much testing but deserves further pursuit, that consenting internet users at some risk for stroke would request continuous surveillance, much like standard computer antivirus programs, to detect an impending stroke so as to allow its prevention.

## Abbreviations

AUC: area under the receiver operating characteristic curve
ROC: receiver operating characteristic
